# Design of upper limb muscle strength assessment system based on surface electromyography signals and joint motion

**DOI:** 10.3389/fneur.2024.1470759

**Published:** 2024-12-13

**Authors:** Siqi Wang, Wei Lai, Yipeng Zhang, Junyu Yao, Xingyue Gou, Hui Ye, Jun Yi, Dong Cao

**Affiliations:** ^1^School of Medical Information Engineering, Guangzhou University of Chinese Medicine, Guangzhou, Guangdong, China; ^2^School of Medical Information Engineering, Guangdong Pharmaceutical University, Guangzhou, Guangdong, China

**Keywords:** upper limb movement disorders, surface electromyographic signals, feature extraction, regression prediction, feature importance, muscle strength assessment

## Abstract

**Purpose:**

This study aims to develop a assessment system for evaluating shoulder joint muscle strength in patients with varying degrees of upper limb injuries post-stroke, using surface electromyographic (sEMG) signals and joint motion data.

**Methods:**

The assessment system includes modules for acquiring muscle electromyography (EMG) signals and joint motion data. The EMG signals from the anterior, middle, and posterior deltoid muscles were collected, filtered, and denoised to extract time-domain features. Concurrently, shoulder joint motion data were captured using the MPU6050 sensor and processed for feature extraction. The extracted features from the sEMG and joint motion data were analyzed using three algorithms: Random Forest (RF), Backpropagation Neural Network (BPNN), and Support Vector Machines (SVM), to predict muscle strength through regression models. Model performance was evaluated using Root Mean Squared Error (*RMSE*), R-Square (*R*^2^), Mean Absolute Error (*MAE*), and Mean Bias Error (*MBE*), to identify the most accurate regression prediction algorithm.

**Results:**

The system effectively collected and analyzed the sEMG from the deltoid muscles and shoulder joint motion data. Among the models tested, the Support Vector Regression (SVR) model achieved the highest accuracy with an *R*^2^ of 0.8059, *RMSE* of 0.2873, *MAE* of 0.2155, and *MBE* of 0.0071. The Random Forest model achieved an *R*^2^ of 0.7997, *RMSE* of 0.3039, *MAE* of 0.2405, and *MBE* of 0.0090. The BPNN model achieved an *R*^2^ of 0.7542, *RMSE* of 0.3173, *MAE* of 0.2306, and *MBE* of 0.0783.

**Conclusion:**

The SVR model demonstrated superior accuracy in predicting muscle strength. The RF model, with its feature importance capabilities, provides valuable insights that can assist therapists in the muscle strength assessment process.

## 1 Introduction

Stroke is a prevalent neurological disorder that significantly diminishes the quality of life for affected individuals. Approximately 40% of stroke survivors experience motor impairments (1), with upper limb dysfunction being particularly common. Such impairments lead to challenges in daily activities and work, significantly reducing the quality of life and independence of these individuals. Early rehabilitation is crucial in recovering upper limb function post-stroke (2–4), aiming to maximize functional restoration and enhance overall quality of life. Prior to initiating rehabilitation, a comprehensive assessment of the upper limb is essential to determine the extent of impairment and to monitor rehabilitation progress (5). These assessments provide a scientific foundation for developing rehabilitation plans, evaluating treatment outcomes, and predicting prognosis, making effective stroke rehabilitation assessment a critical component of stroke care.

Currently, the most commonly used clinical methods for evaluating post-stroke upper limb function are traditional scale-based assessments (6), which primarily rely on the subjective evaluations of rehabilitation therapists. For instance, Katia demonstrated that the Fugl-Meyer Assessment can infer motor performance and movement quality in individuals with varying severities of chronic stroke. Li utilized employed the Brunnstrom Assessment to grade stroke patients, while Shamay used scales such as the Wolf Motor Function Test (WMFT) and the Upper-Body Dressing Scale (UBDS) for the psychological measurement of upper limb function in stroke patients (7–9). Although these scale-based assessments offer standardization, repeatability, and multidimensional evaluation, they are also limited by subjectivity, restricted sensitivity, and significant time and resource consumption.

With advancements in medical technology, modern devices for upper limb function assessment now include wearable sensors and upper limb rehabilitation robots (10). Wearable sensors comprise surface electromyographic (sEMG) sensors, inertial measurement units (IMUs), and more.

Muscles control the movement of the body's limbs, and muscle strength is defined as the maximum force muscles that can exert under specific conditions (11). Muscle strength is typically measured indirectly through methods like sEMG and inverse kinematics (12). sEMG is used to detect the electrical currents generated by muscle contractions during neuromuscular activity, which helps analyze muscle function and assess a patient's muscle condition. sEMG sensors measure the voltage changes between two points on the muscle, capturing muscle activity using electrodes (13, 14). These sensors are non-invasive, significantly reducing infection risk, and are widely used in rehabilitation medicine, movement analysis, and muscle fatigue studies (15). For example, Hsu used EMG sensors to study the sequence of muscle contractions in stroke patients transitioning from sitting to standing and Bandini highlighted the importance of the co-contraction index of sEMG in evaluating the clinical motor performance of stroke patients (16, 17).

Inertial Measurement Units (IMUs) are wearable sensors composed of gyroscopes, accelerometers, and magnetometers. They offer advantages such as portability, low cost, and space efficiency, making them widely used in upper limb movement assessment models (18). For example, researchers have employed 9-axis motion sensors to measure 23 components of the Fugl-Meyer Upper Extremity Assessment. Other studies have used wearable inertial sensors and end-effector robots for precise motion tracking in rehabilitation therapy to evaluate feedback support and rehabilitation outcomes. Sensors with IMUs have been used to collect elbow inertia data, employing machine learning algorithms like random forests for spasticity assessment. Additionally, the MPU6050 inertial sensor has been used to measure upper limb movement direction, with results compared to hand movement trajectories recorded by Kinect sensors (19–22).

In clinical research experiments, sEMG sensors and IMU sensors are often used together for rehabilitation assessment. For instance, Mahmoud et al. combined inertial measurement units, Kinect sensors, and sEMG sensors with occupational therapy to evaluate upper limb function in post-stroke patients. Similarly, Pan et al. assessed upper limb motor function in stroke patients using inertial and sEMG sensors (23, 24). Objective assessment of data collected by sensors requires advanced data processing techniques, often involving machine learning (25). Machine learning technologies have continuously advanced the integration of engineering and medicine over the past few years, particularly in areas such as medical diagnostics and neural system regulation and classification (26–30). Modern algorithms like Random Forest and Support Vector Machines often outperform earlier methods (31, 32). When applied to muscle signals, these algorithms have been used for classifying movements and detecting muscle activity anomalies (30, 33, 34). Additionally, hybrid deep networks combining Long Short-Term Memory (LSTM) and Convolutional Neural Networks (CNN) have achieved an average accuracy of 80% in automatically recognizing Brunnstrom stages III, IV, and V (35).

This study utilizes a surface electromyographic signal acquisition system and the MPU6050 six-axis motion sensor to collect sEMG and motion data during upper limb movements. Regression prediction models are then established using Random Forest (RF), Backpropagation Neural Network (BPNN), and Support Vector Machine (SVM) machine learning methods. The model's performance is evaluated using metrics such as the R-Square (*R*^2^), Root Mean Squared Error (*RMSE*), Mean Absolute Error (*MAE*), and Mean Bias Error (*MBE*). *R*^2^ assesses the model's explanatory power, indicating how well it captures data variability. *RMSE* and *MAE* provide measures of error, helping to understand the model's accuracy. *MBE* reveals systematic bias, identifying areas for improvement. These four evaluation metrics allow for a multidimensional assessment of model performance, enhancing our understanding and guiding future improvements. They also help identify the most optimal algorithm for upper limb muscle strength assessment.

## 2 Materials and methods

**Figure 1:**
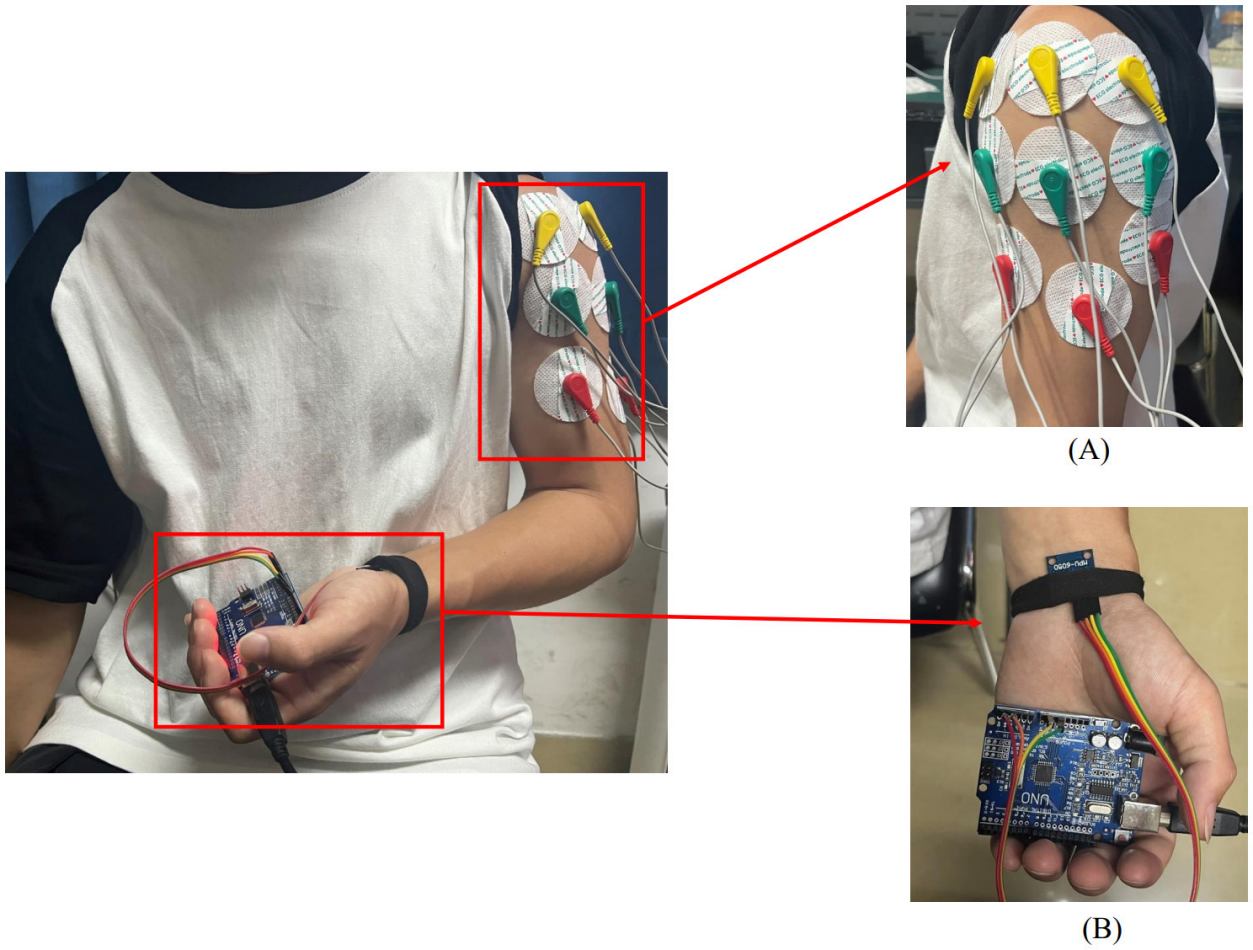
Placement of upper limb testing devices. **(A)** sEMG sensors are placed on the anterior, middle, and posterior deltoid muscles. **(B)** MPU6050 sensor is placed at the distal end of the limb.

**Figure 2:**
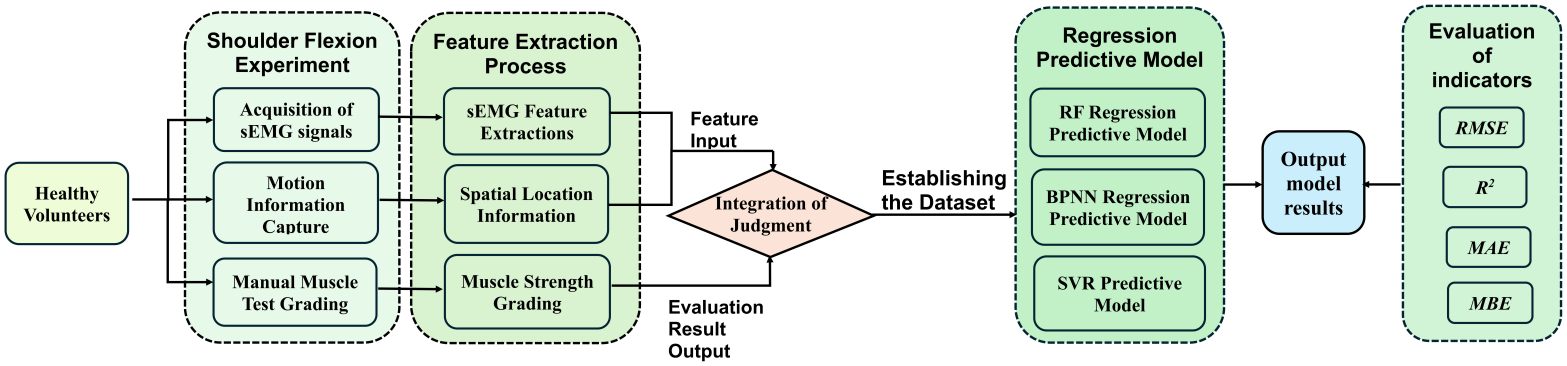
A method for upper limb muscle strength regression prediction based on sEMG and motion capture. RF, Random forest; BPNN, backpropagation neural network; SVR, support vector regression; *RMSE*, root mean squared error; *R*^2^, R-square; *MAE*, mean absolute error; *MBE*, mean bias error.

In this study, a left shoulder flexion test was carried out on healthy volunteers to capture sEMG signals from the anterior, middle, and posterior deltoid muscles, along with motion capture data of the left upper limb. The arrangement of the muscle sensors and MPU6050 sensor is detailed in Figure 1. Following data collection, feature extraction was executed, and the participants' muscle strength was documented utilizing a manual muscle testing scale. Subsequently, machine learning regression models were established to comprehensively assess the level of shoulder flexion strength. The models' performance was evaluated using four key metrics: *RMSE*, *R*^2^, *MAE*, and *MBE*. The outlined research methodology is depicted in Figure 2.

### 2.1 sEMG acquisition and analysis

In the process of upper limb movement, capturing muscle changes is essential. Besides feeling muscle contractions through touch, sEMG sensors can be used to collect signals generated during movement.

#### 2.1.1 sEMG signal acquistion system

**Figure 3:**
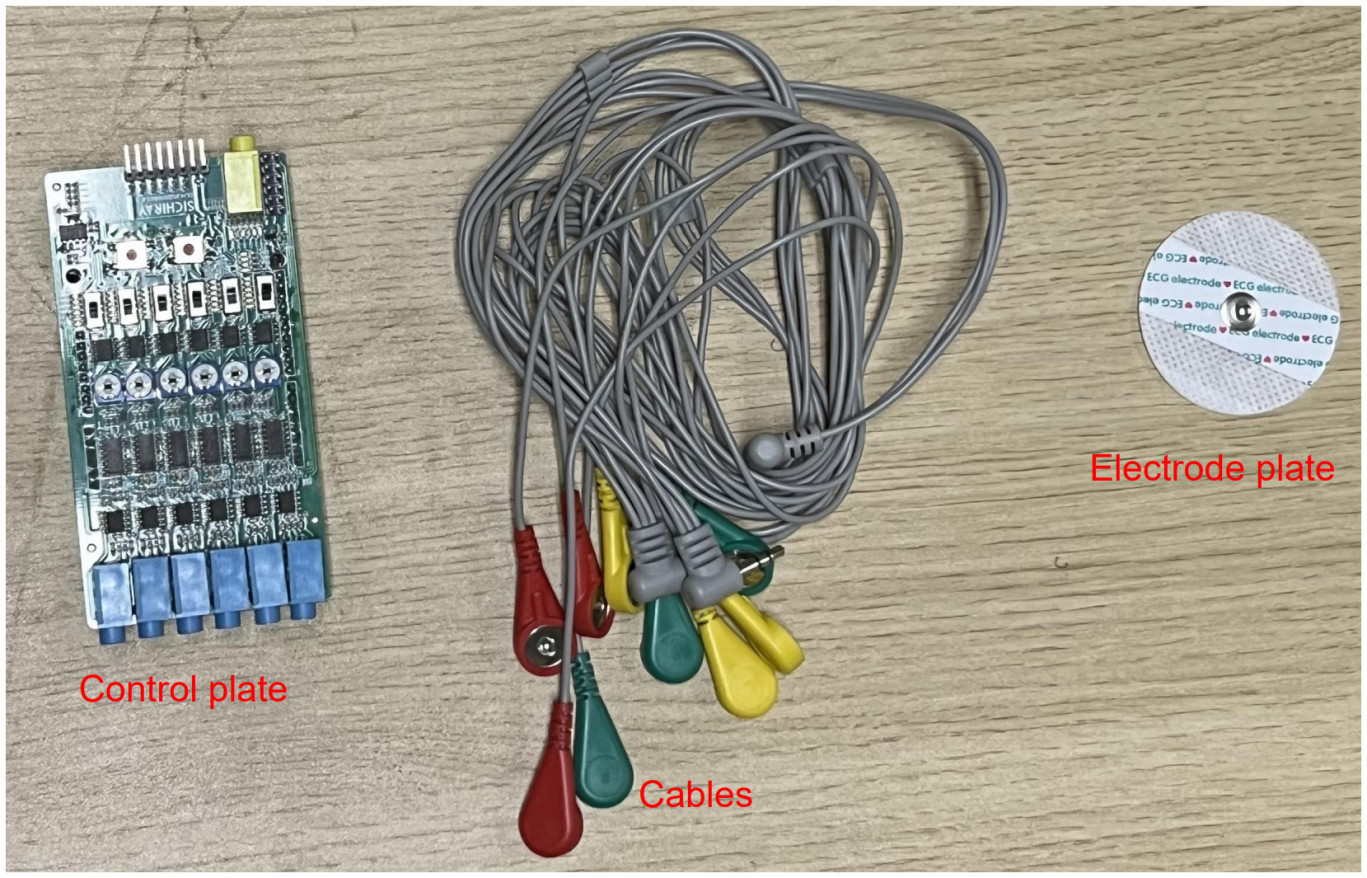
sEMG acquisition device.

The electromyography data was collected using a six-channel EMG sensor device, with a sampling frequency ranging from 1,000 Hz to 5,0000 Hz. After testing, it was found that a sampling frequency of 10,000 Hz minimized environmental noise interference. Thus, this study set the final sampling frequency at 10,000 Hz. The setup of the acquisition device is shown in Figure 3.

In this experiment, shoulder flexion is specified as the motion. The deltoid muscle is one of the main muscles of the shoulder joint, responsible for various movements such as flexion, abduction, and adduction of the shoulder joint. During shoulder flexion, activation of the anterior bundle of the deltoid muscle is most significant. Additionally, the middle and posterior bundles of the deltoid muscle play a supportive role in the stability and support of the shoulder joint. Selecting the anterior, middle, and posterior bundles of the deltoid muscle as the source of sEMG signals allows for a more comprehensive capture of the muscle activity during shoulder flexion, leading to a better understanding of the interaction and coordination among muscles during shoulder joint movements. Therefore, in this experiment, the anterior deltoid, middle deltoid, and posterior deltoid were chosen as the primary data sources for sEMG by analyzing upper limb joint movements. The placement of the EMG electrodes for testing is illustrated in Figure 1A, where the red electrode line indicates the calibration reference point typically positioned at the muscle edge. The yellow and green electrode lines, utilized for sEMG collection, are conventionally placed at the muscle belly. The study focused on shoulder flexion movement of the upper limb and captured the neuromuscular activity of the three deltoid muscle groups during motion.

#### 2.1.2 sEMG preprocessing and feature extraction

**Figure 4:**
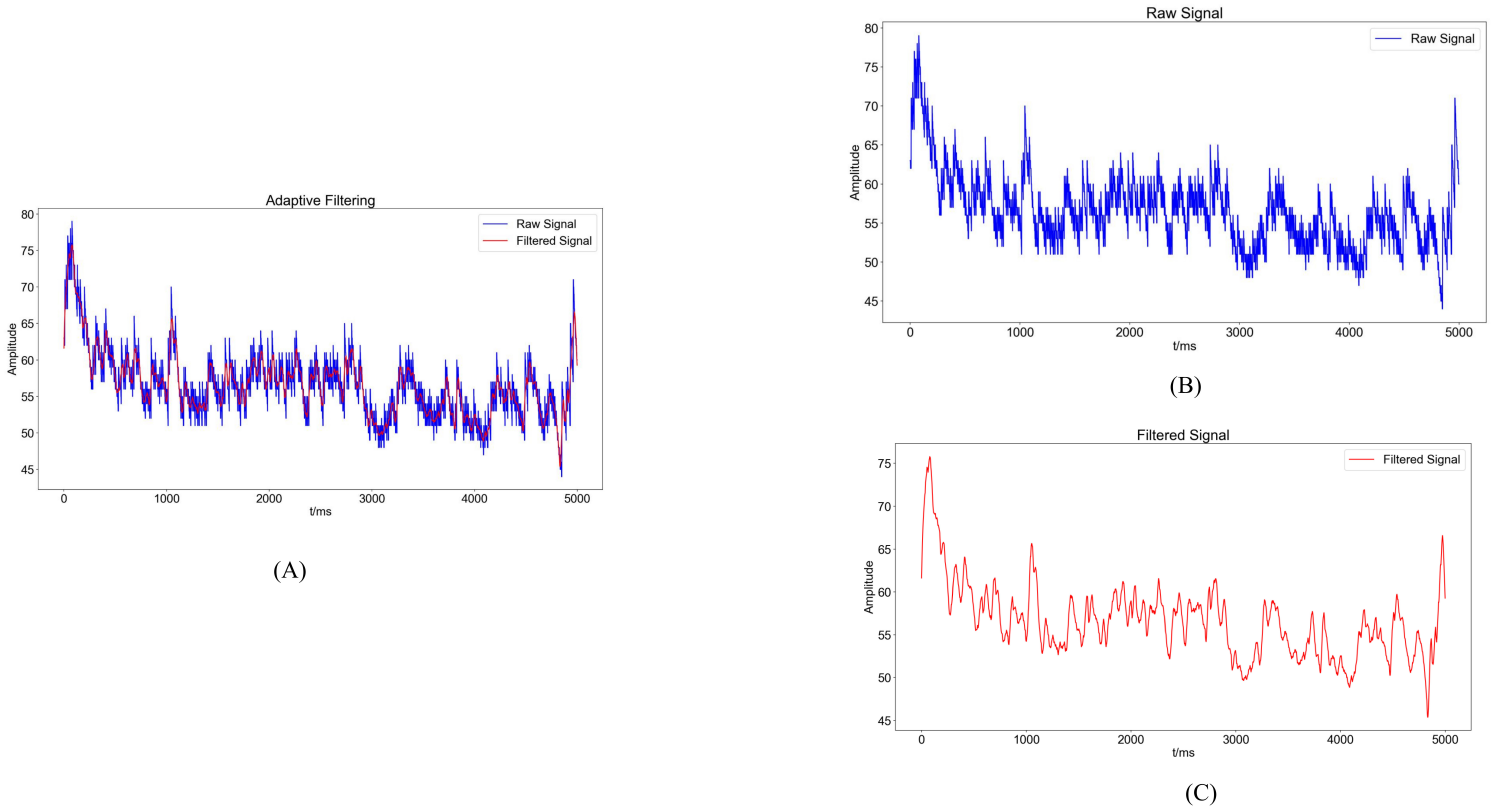
Noise reduction processing results. **(A)** Comparison graph of original sEMG (blue) and sEMG after noise reduction with the Savitzky-Golay filter (red). **(B)** Blue waveform represents the variation of the original sEMG. **(C)** Red waveform represents the sEMG after noise reduction with the Savitzky-Golay filter.

sEMG signals are generated from the combination of electrical potentials produced by multiple motor units beneath the skin's surface. Consequently, they are susceptible to noise interference and signal contamination during the data acquisition phase. Utilizing appropriate filtering algorithms is crucial to eliminate noise from various frequency bands (36). In this experiment, a Savitzky-Golay filter was utilized for noise reduction processing. The Savitzky-Golay filter is a type of digital filter that can enhance data accuracy without altering the signal trend or width. Its advantage lies in preserving the characteristic information of the signal while exhibiting good noise resistance. Therefore, in this experiment, the collected raw electromyography signals were denoised using a Savitzky-Golay filter. The key parameters set for the filter during application include window length and polyorder. Here, window length signifies the number of data points considered by the filter in each smoothing computation. In this study, the window length is set to 51, indicating that the filter will consider 51 data points or 51 milliseconds of time for smoothing operations. Polyorder denotes the order of the polynomial used to fit the data in each window. In this experiment, the polyorder is set to 3, signifying that a third-degree polynomial will be utilized for fitting the data in each window. The results post-completion are depicted in Figure 4.

The present study delves into the analysis of sEMG signals by incorporating four time-domain features: Root Mean Square Value (*RMS*), integral EMG (*iEMG*), Mean Absolute Value (*MAV*), and Variance (*VAR*). Additionally, the feature extracted from the frequency domain includes the median frequency (*MF*).

The *RMS* of the sEMG signifies the average of the amplitudes over a specific time interval. It characterizes the average change in sEMG behavior over time, indicating the energy output during muscle activity and aiding in the assessment of muscle involvement in diverse movements.


(1) $$\begin{array}{l} RMS=\sqrt{\frac{1}{N}\overset{N}{\underset{i=1}{\sum }}x^{2}\left(i\right)} \end{array}$$

On the other hand, the *iEMG* refers to the total area enclosed by the rectified and smoothed sEMG curve within a given time unit. It quantifies the cumulative muscle activity during a designated period, illustrating the temporal intensity fluctuations in the sEMG signal over time.


(2) $$\begin{array}{l} iEMG=\frac{1}{N}\overset{t+T}{\underset{t=1}{\sum }}|EMG\left(t\right)|dt \end{array}$$

In the equation, *EMG*(*t*) represents the collected EMG signal, *t* denotes the time variable, and *T* is the period of the EMG signal.

The *MAV* corresponds to the average of the absolute signal amplitudes within a specific time frame, commonly employed to evaluate muscle contraction intensity and fatigue levels.


(3) $$\begin{array}{l} MAV=\frac{1}{N}\overset{N}{\underset{i=1}{\sum }}|x\left(i\right)| \end{array}$$

In the equation: *N* signifies the number of data points in the collected sEMG, and *x*(*i*) denotes the *i*−*th* data point in the sequence of the signal.

The *VAR* is instrumental in revealing the pattern of sEMG dispersion, illustrating the amplitude changes and extent of variations during movement. It is a standard metric for evaluating the motion signal intensity.


(4) $$\begin{array}{l} VAR=\frac{1}{N}\overset{N}{\underset{i=1}{\sum }}X_{i}^{2} \end{array}$$

In the equation, *N* represents the number of samples, and *X*_*i*_ denotes the amplitude of the sEMG at the *i*−*th* sample.

The *MF* signifies the midpoint frequency of muscle activation during contraction, serving as a reliable indicator of muscle contractile strength and fatigue levels. Typically, the *MF* decreases with prolonged movement durations.


(5) $$\begin{array}{l} MF=\frac{1}{2}\int_{0}^{\infty }PSD\left(f\right)df \end{array}$$

In the equation, *PSD* (Power Spectral Density) denotes the power spectrum of the sEMG, and *df* refers to the sampling frequency.

**Figure 5:**
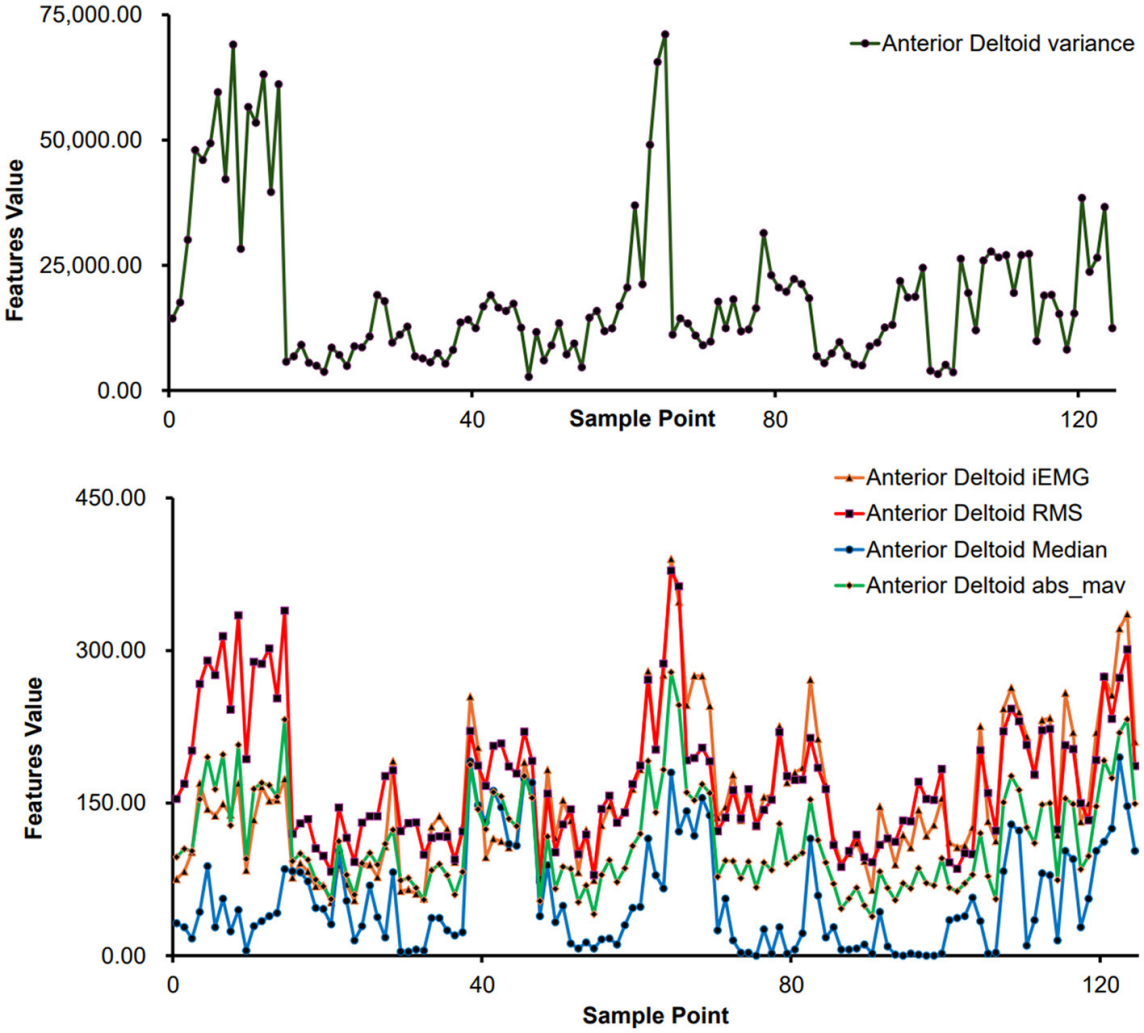
Feature extraction results of different sample points (anterior deltoid muscle).

For sEMG feature extraction, encompassing sample points derived from various movements executed by distinct subjects. Taking the anterior deltoid muscle as an example, the feature values for each sample point are illustrated in Figure 5. Notably, Figure 5 demonstrates significant variances in feature values across different movements, reflecting individual differences in physical conditions and ensuring the data's authenticity.

### 2.2 Inertial sensor acquisition and analysis

During the upper limb rehabilitation process after a stroke, shoulder joint movements often require compensations as patients may move their scapula or trunk. Preventing compensations during the rehabilitation exercises can enhance treatment effectiveness, as compensations can lead to uncertainty in joint movement angle measurements. This issue of accurately measuring joint movement angles can be addressed by using biofeedback methods such as inertial sensor units.

#### 2.2.1 MPU6050 acquisition device

The MPU6050 is a six-degree-of-freedom inertial sensor that integrates a three-axis gyroscope and a three-axis accelerometer (37). The sensor module utilized in this setup comprises the MPU6050 and Arduino UNO. An orientation calculation algorithm devised with the Arduino IDE software is utilized to assess motion angles and acceleration during movement. During shoulder flexion movements, it is common to maintain the elbow joint in an extended position. The MPU6050 is primarily used to capture angular changes during joint movements and is typically placed at the distal end of the limb, such as the biceps tendon or wrist joint. In this study, the MPU6050 was positioned at the wrist joint as shown in Figure 1B. It was secured using an elastic band to prevent displacement in other directions. Additionally, the connection between the MPU6050 and the UNO board was checked to ensure the stability of signal acquisition.

#### 2.2.2 MPU6050 data acquisition and feature processing

During shoulder joint movement analysis, the MPU6050 sensor is positioned at the distal end of the limb to measure the angular movement of the shoulder joint while maintaining a fixed shoulder position. In this study, the data analyzed consisted primarily of three-axis acceleration, three-axis angular velocity, and three-axis azimuth (yaw, pitch, and roll) obtained from the MPU6050 sensor. The yaw angle refers to rotation around the Z-axis, pitch represents rotation around the Y-axis, and roll signifies rotation around the X-axis. Equipped with three gyroscopes and three accelerometers, the MPU6050 sensor outputs crucial data, including three-axis acceleration (Acc) and three-axis angular velocity (Gyro).

The MPU6050 includes an accelerometer and a gyroscope. The accelerometer calculates roll and pitch angles based on the perceived gravitational acceleration when stationary, with angle calculation dependent on the current position. The gyroscope integrates angular velocity over time intervals to derive incremental angle changes, accumulating these changes onto the previous orientation to obtain a new orientation angle. Therefore, a complementary fusion of the attitude calculated from the accelerometer and gyroscope is performed. The accelerometer estimates object orientation by sensing gravitational acceleration, while the yaw angle, typically used to describe directional changes, represents a rotational angle relative to the horizontal plane. Hence, the gyroscope is essential for capturing yaw angle variations. The overall formula for this integration is as follows:


(6) $$\{\begin{array}{c} roll=roll+(roll_{acc}-roll_{gyro})*K\\ pitch=pitch+(pitch_{acc}-pitch_{gyro})*K\\ yaw=yaw_{gyro} \end{array}$$

In the equation, *K* is a proportional coefficient that allows the readings from the accelerometer and gyroscope to be fused according to a certain weighted proportion, thereby reducing the impact of noise and drift to achieve a more precise attitude estimation. Typically, the *K* value ranges from 0 to 1 and can be adjusted based on practical considerations. For this experiment, a value of 0.2 was chosen.

### 2.3 Prediction model establishment and analysis methods

In this study, we utilized sEMG and motion signals to predict muscle strength recovery using RF, BPNN, and SVM methodologies.

#### 2.3.1 RF regression prediction model

RF is a supervised machine learning algorithm utilized for both classification and regression tasks. This algorithm adopts ensemble learning by combining multiple decision trees to forecast outputs in regression analysis (38). Within the RF algorithm, each decision tree comprises root nodes, intermediate nodes, and leaf nodes, with the path from the root to leaf nodes governed by specific rules. The model's accuracy hinges on the number of decision trees employed, with a bootstrap dataset generated for each tree from the main dataset (39–41). In regression analysis, the RF regression prediction model yields the average of the predicted outputs generated across all decision trees (42).

#### 2.3.2 BPNN regression prediction model

BPNN is a deep neural network trained using the backpropagation algorithm, widely recognized as a mature and extensively employed model in the medical domain (43–45). Comprising an input layer, hidden layers, and an output layer, BPNN derives the inter-layer weights through the iterative process of forward signal propagation and backward error propagation.

#### 2.3.3 SVM regression prediction model

SVM is a supervised learning approach utilized for regression and classification tasks. SVM trains the model using a subset of the training data. In regression contexts, this model is termed Support Vector Regressor (SVR) and excels in handling non-linear, high-dimensional challenges, finding extensive use across clinical, physical, engineering sectors, and other fields (39, 46–48). For regression applications, SVR delineates a “margin band” flanking the linear function to allow deviations until a threshold parameter, ε, is exceeded. Loss computations focus solely on samples outside this band, with the model optimization aimed at minimizing overall loss while maximizing the margin. Introducing slack variables ξ and ξ^*^ quantifies the deviations from the predicted output, with the loss function defined as:


(7) $$\{\begin{array}{c} \underset{w,b,\xi_{i},\xi_{i}^{*}}{min}\frac{1}{2}{\left\|w\right\|}^{2}+C\sum_{i=1}^{m}\left(\xi_{i},\xi_{i}^{*}\right)\\ s.t.\;f(x_{i})-y_{i}\leq \varepsilon +\xi_{i}\\ y_{i}-f(x_{i})\leq \varepsilon +\xi_{i}\\ \xi_{i}\geq 0,\xi_{i}^{*}\geq 0,i=1,2,\ldots ,m \end{array}$$

#### 2.3.4 Evaluation metrics

In assessing the effectiveness of various machine learning models, it is customary to employ evaluation metrics that elucidate the predictive accuracy of the models. This study utilizes four key evaluation metrics to ascertain the most effective regression method among the three algorithms. The first metric considered is the coefficient of determination (*R*^2^), a measure that evaluates the precision of machine learning predictions by providing the squared value of the correlation coefficient *R* (49). Typically, a satisfactory *R*^2^ criterion is set at 0.75, with values exceeding 0.75 indicative of a well-fitted model to the data (46).


(8) $$\begin{array}{l} R^{2}=1-\frac{\overset{n}{\underset{i=1}{\sum }}{\left(Actual\;value_{i}-Predicted\;value_{i}\right)}^{2}}{\overset{n}{\underset{i=1}{\sum }}{\left(Actual\;value_{i}-Average\;of\;outputs\right)}^{2}} \end{array}$$

The second evaluation metric is the *RMSE*, which quantifies the square root of the average squared variance between target and predicted values. A lower *RMSE* signifies enhanced predictive accuracy of the model.


(9) $$\begin{array}{l} RMSE=\sqrt{\frac{\overset{n}{\underset{i=1}{\sum }}{\left(Actual\;value_{i}-Predicted\;value_{i}\right)}^{2}}{n}} \end{array}$$

The third evaluation metric is the *MAE*, which works by averaging the absolute differences between the true target values and the predicted values. A smaller *MAE* indicates higher predictive accuracy of the model.


(10) $$\begin{array}{l} MAE=\frac{1}{n}\overset{n}{\underset{i=1}{\sum }}|Actual\;value_{i}-Predicted\;value_{i}| \end{array}$$

The fourth evaluation metric is the *MBE*, which measures the direction of model errors by calculating the average error between predicted values and actual values. A positive *MBE* indicates that the predicted values are higher than the actual values, while a negative value suggests an underestimation of the actual values. The closer the *MBE* is to zero, the smaller the bias of the predictive model.


(11) $$\begin{array}{l} MBE=\frac{1}{n}\overset{n}{\underset{i=1}{\sum }}Actual\;value_{i}-Predicted\;value_{i} \end{array}$$

In this study, the *R*^2^, *RMSE*, *MAE*, and *MBE* metrics are employed to evaluate and contrast the performance of the machine learning regression models, which are executed utilizing Python.

### 2.4 Experimental data acquisition

#### 2.4.1 Data collection

This experiment recruited 15 healthy volunteers to participate in the study. The participants were physically healthy individuals without limb injuries, cognitive impairments, or adverse habits. The data collection took place at the Key Laboratory of Medical Information Engineering, School of Medical Informatics, Guangzhou University of Chinese Medicine. Prior to the experiment, all participants were familiarized with the experimental protocol and procedures, and they provided informed consent by signing a consent form. The entire experiment was reviewed and approved by the Ethics Committee for Human Biomedical Experiments at Guangdong Provincial Hospital of Traditional Chinese Medicine.

The sEMG was collected using a six-channel EMG muscle electrode sensor, while the motion data was captured using an MPU6050 inertial sensor. According to the physiological structure of the human body, synchronized sEMG were collected from the anterior deltoid, middle deltoid, and posterior deltoid muscles of the left upper limb of each participant, along with the velocity and angular data during the movement of the left upper limb.

Before testing, the participants cleaned the skin around the muscles with 75% alcohol to reduce low-frequency noise caused by electrode movement due to sweat. During the experiment, the participants remained seated with their elbow joints in a fully extended position. They performed shoulder flexion movements of the left upper limb, and each complete shoulder flexion movement was defined as lifting the left upper limb from the midline of the trunk and returning it to the original position. Each participant performed 10 shoulder flexion movements at different angles. During the movements, the participants also underwent manual muscle testing (MMT) with the assistance of a professional rehabilitation therapist, following the MMT muscle grading standards (as shown in Table 1).

**Table 1:** MMT muscle grading standards.

**Level**	**Performance**
0	No muscle contraction
1	Muscle contraction palpable, but no joint movement
2	Movement throughout the full range of motion with gravity eliminated
3	Movement throughout the full range of motion against gravity, but not against resistance
4	Movement against gravity and some resistance
5	Movement against gravity and full resistance

#### 2.4.2 Database setup

In this experiment, a total of 15 healthy participants each performed 10 repetitions of shoulder flexion movements, resulting in 150 data points collected. A dataset was constructed based on the gathered sEMG and motion information, extracting features such as *iEMG*, *RMS*, *MAV*, *VAR*, *MF*, and 24 features collected by the MPU6050 including Gyrox, Gyroy, Gyroz, Accx, Accy, Accz, Pitch, Roll, and Yaw. Each of the 150 data points was characterized by these 24 features. Muscle strength in the participants was assessed using the Manual Muscle Testing (MMT) scale, which categorizes muscle strength from 0 to 5. Rehabilitation therapists evaluated muscle strength based on the movement process combined with the MMT scale, resulting in a muscle strength assessment for each movement of each participant, totaling 150 assessments. Due to factors such as muscle fatigue, a subset of 125 data points was selected for analysis after screening.

The dataset was formed by taking the features as independent variables and the muscle strength as the target variable (dependent variable). The dataset was divided into a 70% training set and a 30% testing set. The regression models were trained using the training set, and the trained models were used to predict the muscle strength on the testing set. Finally, the performance of the models was evaluated using four evaluation metrics: *R*^2^, *RMSE*, *MAE*, and *MBE*. To mitigate the influence of unit differences between variables, a linear transformation function was applied to normalize the variables (50).


(12) $$\begin{array}{l} y=\frac{x-Min_{x}}{Max_{x}-Min_{x}} \end{array}$$

## 3 Results

### 3.1 Experimental results analysis

#### 3.1.1 Results of the RF regression model

**Figure 6:**
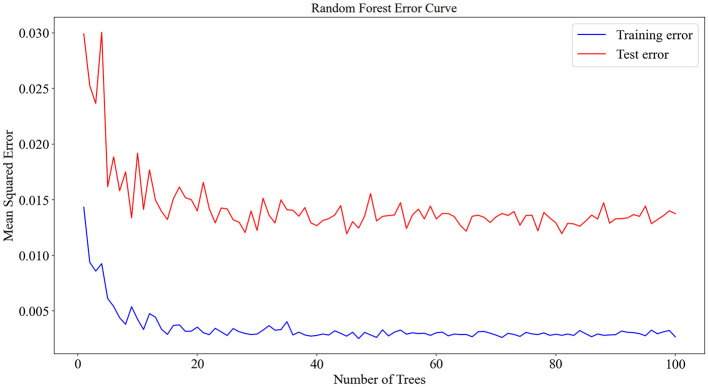
Decision tree error curve for training and testing sets.

**Figure 7:**
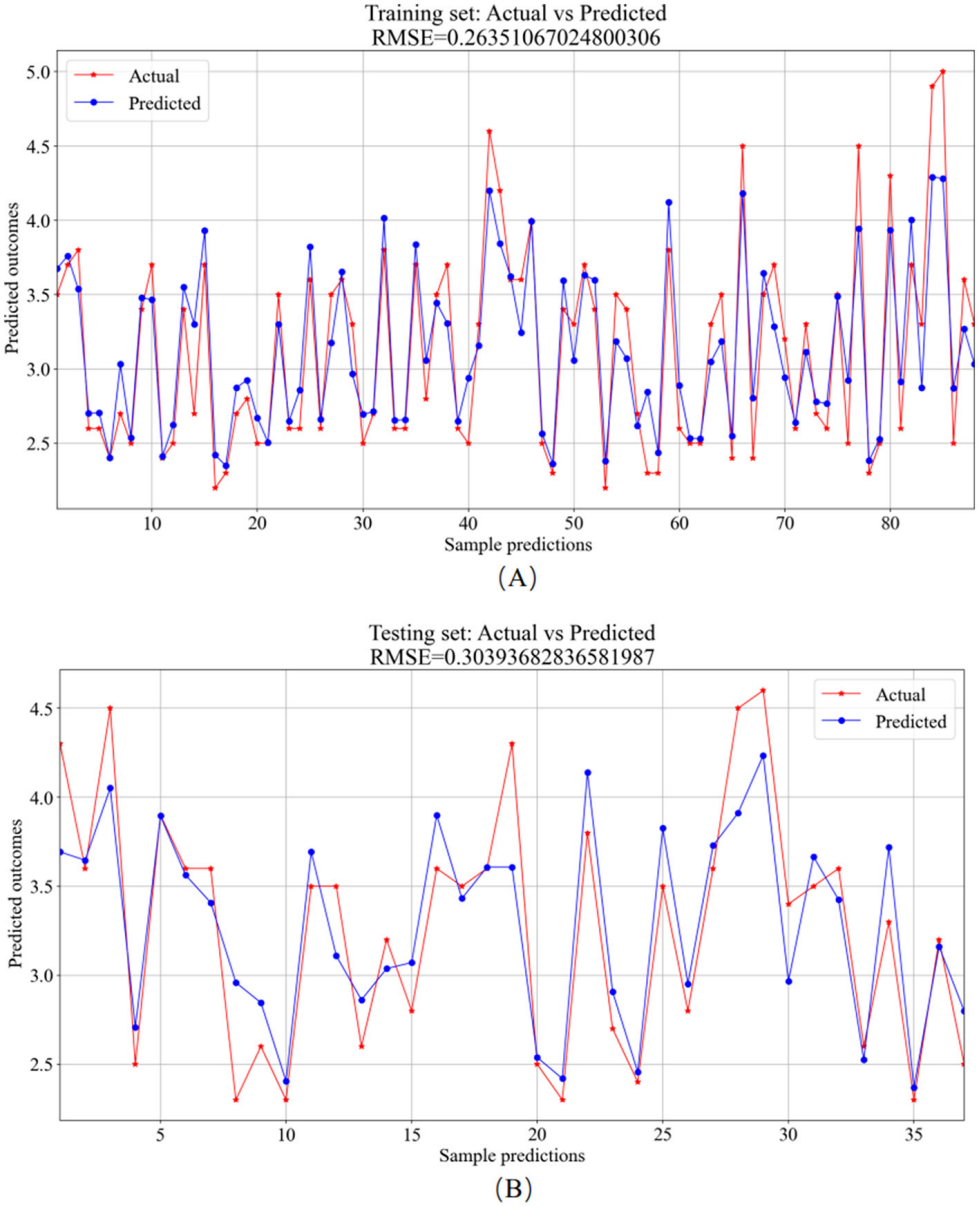
RF model prediction results. **(A)** Training set—predicted values vs. actual values. **(B)** Testing set–predicted values vs. actual values.

**Figure 8:**
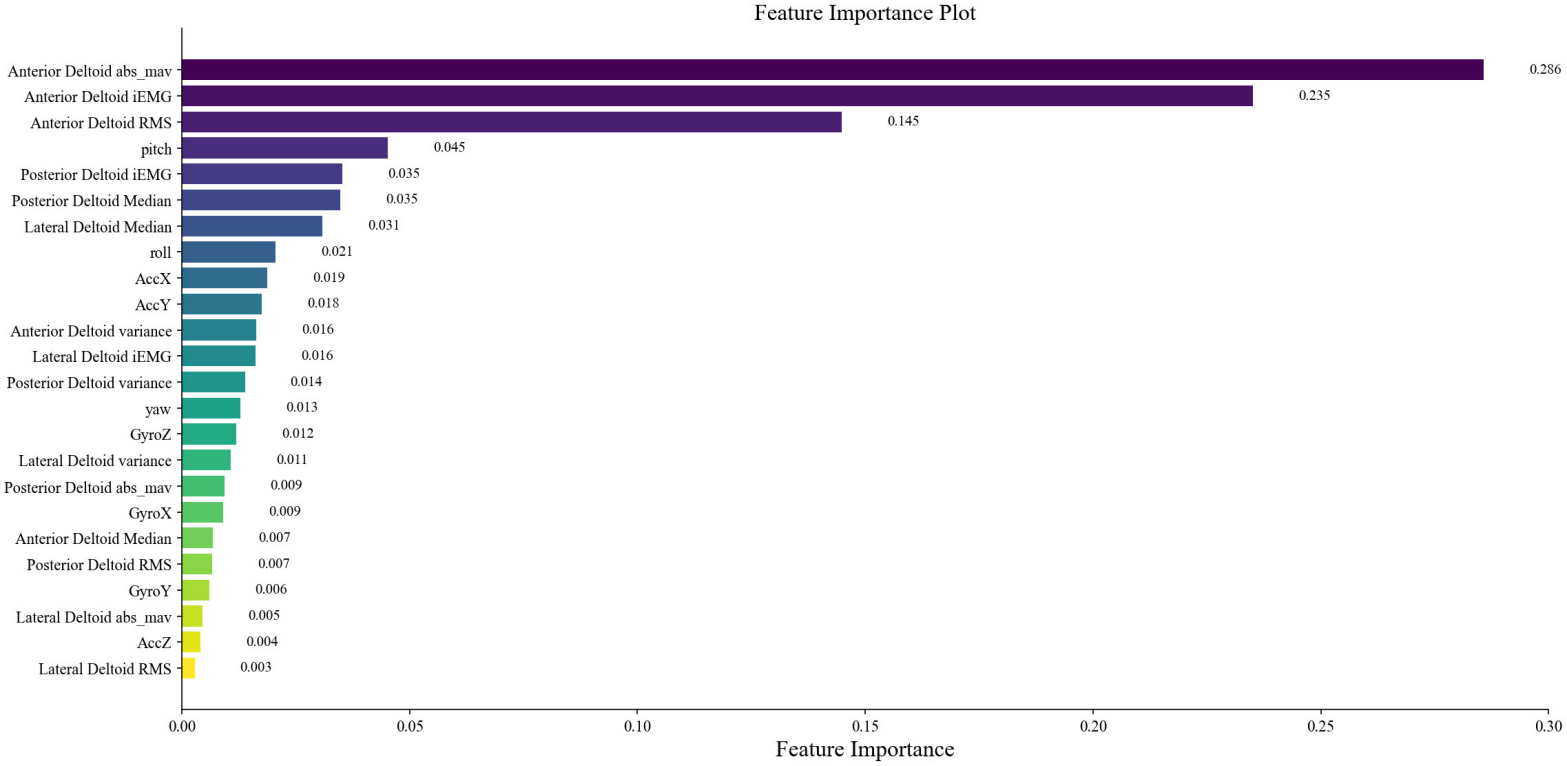
Distribution of feature importance.

The 125 selected data points from this experiment were integrated into a RF model, and the model was fine-tuned by adjusting parameters, focusing on key training parameters such as the number of decision trees and the minimum samples required at each leaf node. The number of decision trees is crucial for enhancing the model's stability and accuracy, albeit at the cost of increased training time. On the other hand, setting a minimum sample requirement at each leaf node helps in reducing model complexity and guarding against overfitting. In this experiment, 100 decision trees and a minimum leaf size of 5 were selected. The RF model was trained and predicted on the training and testing sets, respectively. The results encompassed the decision tree error curve (Figure 6), training set outcomes (Figure 7A), test set predictions (Figure 7B), and feature importance analysis (Figure 8).

The analysis of the error values depicted in Figure 6 reveals a stabilization tendency below 0.02 when the decision tree count ranges between 20 and 100. Notably, exceeding 100 decision trees escalates the computational time significantly. Consequently, to balance between computational efficiency and model performance, a decision tree count of 100 was deemed optimal. At this juncture, the average error on the test set falls below 0.02, signaling robust stability and efficacy. The training set plays a foundational role in nurturing the RF regression model. As evidenced in Figure 7A, the comparison of predicted versus actual values in the training set yielded an *RMSE* of 0.2635. Subsequently, the testing set utilizes the trained model for prediction, as shown in Figure 7B, where the *RMSE* value stands at 0.3039.

The main advantage of RF lies in its capability to conduct feature importance selection. This determination is based on the entire dataset rather than individual training or testing sets, as displayed in the rankings and scores of feature importance in Figure 8. Extracted from sEMG and motion data, 24 features were derived and assessed using the RF model for ensemble learning. Notably, all features exhibit positive correlations with muscle strength, with prominent roles played by features like *RMS*, *iEMG*, *MAV*, and pitch of the anterior deltoid in evaluating shoulder flexion strength. The dominance of anterior deltoid-related features over those linked to the middle and posterior deltoids aligns well with the mechanics of shoulder flexion. Among the offset angles captured by MPU6050, pitch emerges as the pivotal variable. Given that the study predominantly measures shoulder flexion in the left-handed coordinate system, where the humerus rotates around the coronal axis in the sagittal plane (corresponding to the Y-axis rotation of MPU6050) during flexion, pitch assumes vital importance as the primary offset angle. These observations advocate for the relative accuracy of the feature importance outcomes derived from the RF model.

#### 3.1.2 Results of the BPNN regression model

**Figure 9:**
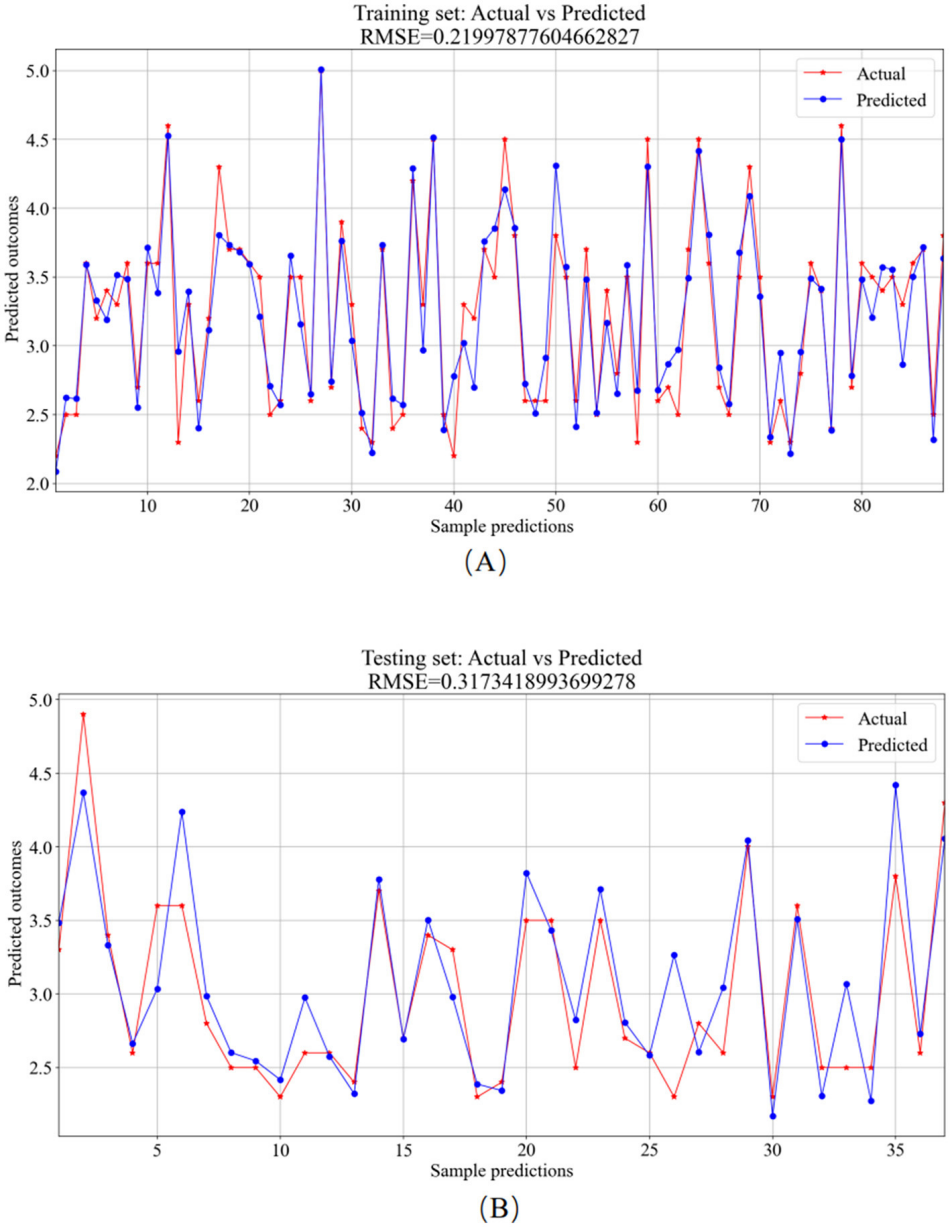
BPNN model prediction results. **(A)** Training set—predicted values vs. actual values. **(B)** Testing set—predicted values vs. actual values.

The 125 selected data points from this experiment were integrated into a BPNN regression model, which was further optimized by adjusting key model parameters. The primary training parameters included the number of neurons in the hidden_layer_sizes, the max_iter, the activation function, tolerance, and initial learning rate. The number of neurons in the hidden_layer_sizes determines the model's complexity and learning capacity, with an excessive number potentially leading to overfitting. The number of max_iter sets the maximum training iterations to prevent overfitting or prolonged training. The choice of activation function defines the output format of neurons. Tolerance helps enhance efficiency by halting training when the loss function change falls below the specified tolerance, preventing unnecessary iterations. The initial learning rate determines the magnitude of weight updates in each iteration, aiding in faster convergence. For this experiment, the model had 5 neurons in the hidden_layer_sizes, 1000 max_iter, “rule” activation function, tolerance set to 1e-6, and an initial learning rate of 0.01. The BPNN regression model was trained and predicted on the training and testing sets, respectively. The results include the training set training results (Figure 9A) and the testing set prediction results (Figure 9B).

The training set was primarily utilized for BP neural network model training. Figure 9A showcases the comparison between predicted and actual values in the training set, resulting in an *RMSE* value of 0.2200. In the testing phase, the trained model was applied for prediction on the test set, depicted in Figure 9B, with an *RMSE* value of 0.3173, showcasing the comparison between predicted and actual values.

#### 3.1.3 Results of the SVR model

**Figure 10:**
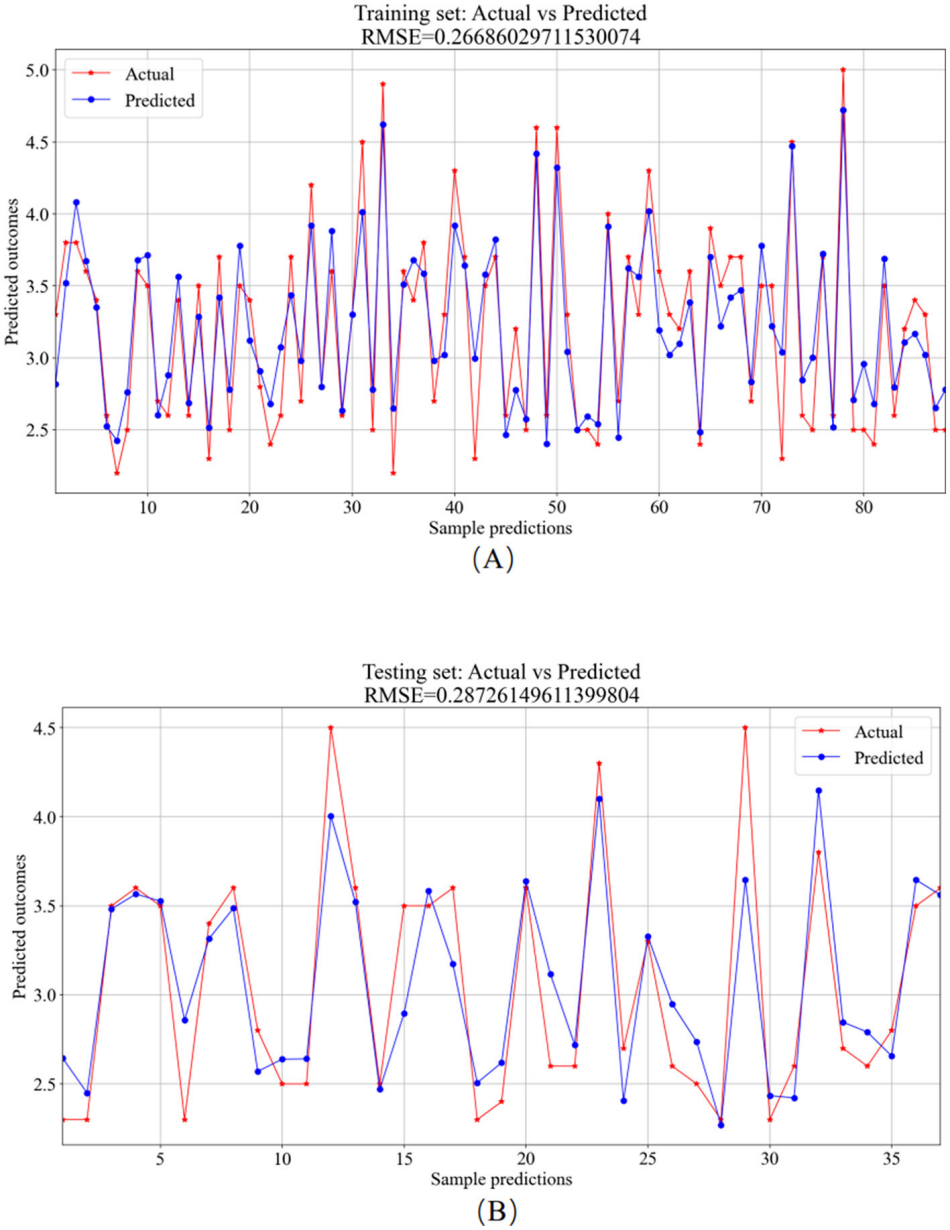
SVR model prediction results. **(A)** Training set—predicted values vs. actual values. **(B)** Testing set—predicted values vs. actual values.

The 125 selected data points from this experiment were integrated into the SVR model, and parameter tuning was performed on the model. The important parameters include the kernel function (kernel) and its parameters, the regularization parameter (C), and the epsilon parameter (epsilon). The kernel function is used to map input features to a high-dimensional space to enable linear separation in non-linear problems. The coefficient gamma of the kernel function controls the influence range of individual training samples. A large gamma value can lead to overfitting, while a small value may result in underfitting. The parameter C governs the model's complexity and tolerance. A higher C value causes the model to focus more on the training data, potentially leading to overfitting. The tolerance parameter is used to define the margin of error allowed by the SVR model during the fitting process. In this experiment, the RBF (Radial Basis Function) kernel function was used with a gamma parameter of 0.1, a regularization parameter (C) of 10, and an epsilon parameter of 0.1. The SVR model was trained and predicted on the training and testing sets, respectively. The results include the training set training results (Figure 10A) and the testing set prediction results (Figure 10B).

The training set served as the primary platform for training the SVR model. Figure 10A provides a visual comparison between the predicted and actual values from the training set, resulting in an *RMSE* value of 0.2669. In contrast, the testing set utilized the trained model for predictions, showcasing the comparative analysis of predicted versus actual values in Figure 10B, with an *RMSE* value of 0.2873.

### 3.2 Regression model comparison

**Figure 11:**
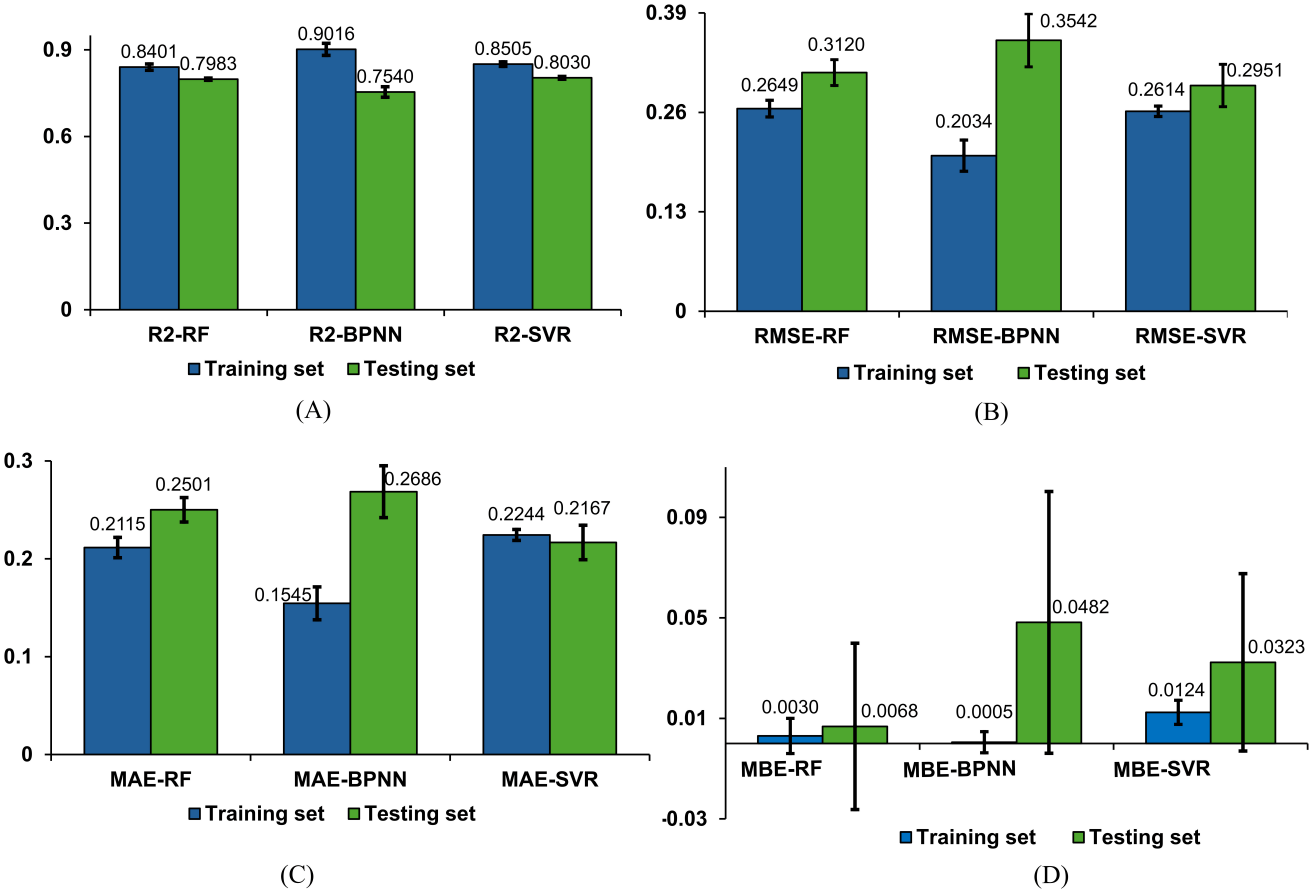
Comparison of regression models. **(A)** Comparison of *R*^2^ results for three models. **(B)** Comparison of *RMSE* results for three models. **(C)** Comparison of *MAE* results for three models. **(D)** Comparison of *MBE* results for three models.

To delve into the performance of machine learning models in detail, we conducted multiple experiments. The average performance metrics (*R*^2^, *RMSE*, *MAE*, and *MBE*) for the test and training sets, along with their standard deviations, are graphically presented in Figures 11A–11D. Additionally, the average values and standard deviations of all evaluation metrics across different models for the test sets, along with the p-values, are displayed in Table 2. From the results in Table 2, it is observed that the *R*^2^ average values for the RF, BPNN, and SVR models on the test sets all exceed 0.75, indicating good fitting performance for all three models. Among them, the SVR model demonstrates relatively superior fitting performance with an *R*^2^ average value of 0.8030. The RF model shows relatively good fitting performance with an *R*^2^ average value of 0.7983, while the BPNN model's fitting performance is comparatively moderate with an *R*^2^ average value of 0.7540.

**Table 2:** Results for four models on the testing set.

**Model**	**Evaluation metrics**			
	*R*^2^ **(mean** ± **SD)**	*RMSE* **(mean** ± **SD)**	*MAE* **(mean** ± **SD)**	*MBE* **(mean** ± **SD)**
BPNN	0.7540 ± 0.0182	0.3542 ± 0.0347	0.2686 ± 0.0365	0.0482 ± 0.0521
RF	0.7983 ± 0.0038	0.3120 ± 0.0169	0.2501 ± 0.0277	0.0068 ± 0.0331
SVR	0.8030 ± 0.0048	0.2951 ± 0.0277	0.2167 ± 0.0176	0.0323 ± 0.0353
P-Value	< 0.001	0.009	0.009	< 0.001

The evaluation metrics for the test sets of the RF, BPNN, and SVR models include *RMSE*, *MAE*, and *MBE*. The SVR model exhibits higher prediction accuracy with an *RMSE* average value of 0.2951 and standard deviation of 0.0277, an *MAE* average value of 0.2167 with a standard deviation of 0.0176, and an *MBE* average value of 0.0323 with a standard deviation of 0.0353. The RF model shows relatively high prediction accuracy with an *RMSE* average value of 0.3120 and standard deviation of 0.0169, an *MAE* average value of 0.2501 with a standard deviation of 0.0125, and an *MBE* average value of 0.0068 with a standard deviation of 0.0331. The BPNN model exhibits prediction accuracy with an *RMSE* average value of 0.3542 and standard deviation of 0.0347, an *MAE* average value of 0.2686 with a standard deviation of 0.0265, and an *MBE* average value of 0.0482 with a standard deviation of 0.0521.

Based on the results of the standard deviations, it can be observed that the standard deviations among the four performance metrics are relatively small, indicating a low level of dispersion. The P-values demonstrate the significance levels of each metric, aiding in evaluating the performance differences among models across different evaluation metrics. According to the results in Table 2, it is believed that there are significant performance differences among the three models, suggesting that a comprehensive assessment should consider all four evaluation metrics in a multi-dimensional approach.

Integrating the four evaluation metrics along with the average values and standard deviations of each evaluation metric across different models, it can be concluded that the SVR and RF regression models outperform the BPNN regression model. Specifically, the SVR model exhibits slight superiority over the RF model in terms of prediction accuracy, indicating that SVR demonstrates better performance and predictive capability for this regression task.

## 4 Discussion

In designing this system, three widely-used machine learning regression models were employed: RF, BPNN, and SVR. Among these, the RF and SVR models demonstrated notable advantages over the BPNN model, each exhibiting distinct strengths. The primary advantage of the RF model is its ability to mitigate the risk of overfitting by integrating multiple decision trees, making it relatively robust to small amounts of noisy data (51). Conversely, the SVR model excels by constructing support vectors and hyperplanes, which enables it to handle non-linear relationships effectively and demonstrate strong generalization capabilities (38). In contrast, the BPNN model requires extensive training and parameter tuning, especially when dealing with complex non-linear relationships, large datasets, and the need to avoid overfitting.

The RF model implemented in the upper limb rehabilitation assessment detailed in this research empowers rehabilitation therapists to target vital muscles in the rehabilitation process by exploiting feature importance. Furthermore, the discernment of compensatory behaviors involving alternate muscles in patients is facilitated through this emphasis on feature importance.

Conversely, the SVR model employed in the upper limb rehabilitation assessment system outlined in this study leverages support vectors and hyperplanes to analyze surface electromyographic signals and joint movements, providing insights for predicting upper limb rehabilitation assessment outcomes.

The experimental findings in this study indicate that the RF model outperformed BPNN in terms of achieving lower *RMSE* and *MBE* on the testing set, showcasing superior prediction accuracy for RF. Conversely, the RF model exhibited slightly higher *MAE* compared to BPNN on the testing set. This discrepancy highlights the distinct strengths of RF and BPNN in handling diverse dataset characteristics; RF excels in managing complex non-linear relationships, whereas BPNN demonstrates advantages in addressing intricate patterns and correlations. Given the dataset's abundance of non-linear relationships, RF showcased enhanced data fitting capabilities, resulting in reduced *RMSE* and *MBE*. However, the *MAE* metric, because it does not consider error direction, might lead to higher deviations between RF's predictions and actual values. This underscores the significance of incorporating multiple evaluation metrics when selecting and assessing models to ensure a comprehensive evaluation of their performance and adaptability.

The implications of this research are particularly pertinent for individuals with upper limb impairments post-clinical stroke. By utilizing the device to gather pertinent data on upper limb movements and utilizing appropriate models to predict muscle strength outcomes, rehabilitation therapists can craft personalized and precise rehabilitation strategies, providing a valuable framework for tailored and precise rehabilitation strategies.

However, this study is subject to several limitations. Firstly, the study cohort comprised healthy adults aged 20–28, warranting future investigations considering demographic variations such as age, history of skeletal joint diseases, and geriatric conditions. If conditions permit, further data collection on stroke patients' rehabilitation training and assessment will be conducted to enhance the database. Secondly, the research solely concentrated on electromyographic and joint activity data collection during shoulder flexion movements, neglecting multi-joint and multi-directional movements. Thus, further enhancements are imperative to refine the predictive accuracy of the proposed method for assessing upper limb muscle strength.

## 5 Conclusion

In conclusion, this study introduces an upper limb rehabilitation muscle strength assessment system based on parameters like motion speed, angular displacement, and data acquired through sEMG and MPU6050. Addressing the limitations of exclusive reliance on sEMG for motion recognition, the system emphasizes the analysis of muscle strength levels during shoulder flexion. By utilizing sEMG and motion capture methodologies, machine learning regression models are developed to forecast muscle strength levels. Conducting experiments on 15 participants involving upper limb shoulder flexion movements validated the fitting performance and accuracy of the RF, BPNN, and SVR regression models. Among these models, the SVR model demonstrates relatively superior overall performance, followed by the RF regression prediction model. Both SVR and RF exhibit significantly higher accuracy compared to BPNN. The study outcomes underscore the utility of the proposed model, utilizing sEMG and joint motion data, in guiding shoulder flexion movements and rehabilitation interventions. This framework offers valuable guidance to therapists in devising individualized rehabilitation strategies, thus furnishing both theoretical and technical underpinnings for personalized care plans in the future.

## Data Availability

The raw data supporting the conclusions of this article will be made available by the authors, without undue reservation.

## References

[1] Wang A Tian X Jiang D Yang C Xu Q Zhang Y. Rehabilitation with brain-computer interface and upper limb motor function in ischemic stroke: a randomized controlled trial. Medicine. (2024) 5:559–69. 10.1016/j.medj.2024.02.014 38642555

[2] Tang Z Liu T Liu Y Han K Su W Zhao J. Different doses of intermittent theta burst stimulation for upper limb motor dysfunction after stroke: a study protocol for a randomized controlled trial. Front Neurosci. (2023) 17:1259872. 10.3389/fnins.2023.1259872 37869516 PMC10585036

[3] Fernández-Solana J Álvarez-Pardo S Moreno-Villanueva A Santamaria-Pelíaez M González-Bernal JJ Vélez-Santamaría R. Efficacy of a rehabilitation program using mirror therapy and cognitive therapeutic exercise on upper limb functionality in patients with acute stroke. Healthcare. (2024) 12:569. 10.3390/healthcare12050569 38470680 PMC10931296

[4] Sharififar S Shuster JJ Bishop MD. Adding electrical stimulation during standard rehabilitation after stroke to improve motor function. A systematic review and meta-analysis. Ann Phys Rehab Med. (2018) 61:339–44. 10.1016/j.rehab.2018.06.005 29958963

[5] Bai J Li G Lu X Wen X. Automatic rehabilitation assessment method of upper limb motor function based on posture and distribution force. Front Neurosci. (2024) 18:1362495. 10.3389/fnins.2024.1362495 38440394 PMC10909926

[6] Deb S Islam MF Rahman S Rahman S. Graph convolutional networks for assessment of physical rehabilitation exercises. IEEE Trans Neural Syst Rehab Eng. (2022) 30:410–9. 10.1109/TNSRE.2022.3150392 35139022

[7] Rech KD Salazar AP Marchese RR Schifino G Cimolin V Pagnussat AS. Fugl-Meyer assessment scores are related with kinematic measures in people with chronic hemiparesis after stroke. J Stroke Cerebrovasc Dis. (2020) 29:1044634. 10.1016/j.jstrokecerebrovasdis.2019.104463 31740027

[8] Li R Zheng S Zhang Y Zhang H Du L Cheng L. Quantitative assessment of thenar to evaluate hand function after stroke by Bayes discriminant. BMC Musculoskel Disord. (2023) 24:682. 10.1186/s12891-023-06789-w 37644487 PMC10463400

[9] Shamay S Peiming C Chang HS Wing-Kiu C Tsz-Ho K Yunon L. Psychometric properties of upper-body dressing scale in people with stroke. J Rehabil Med. (2023) 55:5766. 10.2340/jrm.v55.5766 37073768 PMC10161440

[10] Simba na EDO Baeza PSH Huete AJ Balaguer C. Review of automated systems for upper limbs functional assessment in neurorehabilitation. IEEE Access. (2019) 7:32352–67. 10.1109/ACCESS.2019.2901814

[11] Bohannon RW. Considerations and practical options for measuring muscle strength: a narrative review. Biomed Res Int. (2019) 2019:8194537. 10.1155/2019/8194537 30792998 PMC6354207

[12] Borbély BJ Szolgay P. Real-time inverse kinematics for the upper limb: a model-based algorithm using segment orientations. Biomed Eng. (2017) 16:1–29. 10.1186/s12938-016-0291-x 28095857 PMC5240469

[13] Chen Z Wang Q Bi Y Lin J Yang W Deng C. Analyzing human muscle state with flexible sensors. J Sensors. (2022) 2022:5227955. 10.1155/2022/5227955

[14] Wu YD Ruan SJ Lee YH. An ultra-low power surface EMG sensor for wearable biometric and medical applications. Biosensors. (2021) 11:411. 10.3390/bios11110411 34821627 PMC8615488

[15] Huang P Wang H Wang Y Liu Z Samuel OW Yu M. Identification of upper-limb movements based on muscle shape change signals for human-robot interaction. Comput Math Methods Med. (2020) 2020:5694265. 10.1155/2020/5694265 32351614 PMC7178526

[16] Bandini V Carpinella I Marzegan A Jonsdottir J Frigo CA Avanzino L. Surface-electromyography-based co-contraction index for monitoring upper limb improvements in post-stroke rehabilitation: a pilot randomized controlled trial secondary analysis. Sensors. (2023) 23:7320. 10.3390/s23177320 37687775 PMC10490112

[17] Hsu WC Chang CC Lin YJ Yang FC Lin LF Chou KN. The use of wearable sensors for the movement assessment on muscle contraction sequences in post-stroke patients during sit-to-stand. Sensors. (2019) 19:657. 10.3390/s19030657 30736269 PMC6387101

[18] Meng L Chen M Li B He F Xu R Ming D. An inertial-based upper-limb motion assessment model: performance validation across various motion tasks. IEEE Sens J. (2023) 23:7168–77. 10.1109/JSEN.2022.3233344

[19] Ueyama Y Takebayashi T Takeuchi K Yamazaki M Hanada K Okita Y. Attempt to make the upper-limb item of objective fugl-meyer assessment using 9-axis motion sensors. Sensors. (2023) 23:5213. 10.3390/s23115213 37299941 PMC10255665

[20] Passon A Schauer T Seel T. Inertial-robotic motion tracking in end-effector-based rehabilitation robots. Front Robotics and AI. (2020) 7:554639. 10.3389/frobt.2020.554639 33501318 PMC7806092

[21] Kim JY Park G Lee SA Nam Y. Analysis of machine learning-based assessment for elbow spasticity using inertial sensors. Sensors. (2020) 20:1622. 10.3390/s20061622 32183281 PMC7146614

[22] Acharya A Bhat S Kanthi M. A comparative analysis of two approaches for estimation of upper limb orientation using inertial and kinect sensors. Adv Elect Comp Eng. (2022) 22:4. 10.4316/AECE.2022.0401022644120

[23] Pan B Huang Z Jin T Wu J Zhang Z Shen Y. Motor function assessment of upper limb in stroke patients. J Healthc Eng. (2021) 2021:6621950. 10.1155/2021/6621950 33708365 PMC7932780

[24] Mahmoud SS Cao Z Fu J Gu X Fang Q. Occupational therapy assessment for upper limb rehabilitation: a multisensor-based approach. Front Digital Health. (2021) 3:784120. 10.3389/fdgth.2021.784120 34977858 PMC8718516

[25] Chung CR Su MC Lee SH Wu EHK Tang LH Yeh SC. An intelligent motor assessment method utilizing a bi-lateral virtual-reality task for stroke rehabilitation on upper extremity. IEEE J Transl Eng Health Med. (2022) 10:1–11. 10.1109/JTEHM.2022.3213348 36457894 PMC9704741

[26] Wang S Hu Y Shen Y Li H. Classification of diffusion tensor metrics for the diagnosis of a myelopathic cord using machine learning. Int J Neural Syst. (2018) 28:1750036. 10.1142/S0129065717500368 28830310

[27] Górriz JM Ramírez J Segovia F Martínez FJ Lai MC Lombardo MV. A machine learning approach to reveal the neurophenotypes of autisms. Int J Neural Syst. (2019) 29:1850058. 10.1142/S0129065718500582 30782022

[28] Gaur P McCreadie K Pachori RB Wang H Prasad G. Tangent space features-based transfer learning classification model for two-class motor imagery brain-computer interface. Int J Neural Syst. (2019) 29:1950025. 10.1142/S0129065719500254 31711330

[29] Zhang X Foderaro G Henriquez C Ferrari S A. scalable weight-free learning algorithm for regulatory control of cell activity in spiking neuronal networks. Int J Neural Syst. (2018) 28:1750015. 10.1142/S0129065717500150 28270025

[30] Gómez-Vilda P Gómez-Rodellar A Vicente JMF Mekyska J Palacios-Alonso D Rodellar-Biarge V. Neuromechanical modelling of articulatory movements from surface electromyography and speech formants. Int J Neural Syst. (2019) 29:1850039. 10.1142/S0129065718500399 30409059

[31] Yu B Wang H Shan W Yao B. Prediction of bus travel time using random forests based on near neighbors. Comp-Aided Civil Infrastruct Eng. (2018) 33:333–50. 10.1111/mice.12315

[32] Zhang J Xiao M Gao L Chu S. Probability and interval hybrid reliability analysis based on adaptive local approximation of projection outlines using support vector machine. Comp-Aided Civil Infrastruct Eng. (2019) 34:991–1009. 10.1111/mice.12480

[33] Burns A Adeli H Buford JA. Upper limb movement classification via electromyographic signals and an enhanced probabilistic network. J Med Syst. (2020) 44:176. 10.1007/s10916-020-01639-x 32829419

[34] Dai C Hu X. Extracting and classifying spatial muscle activation patterns in forearm flexor muscles using high-density electromyogram recordings. Int J Neural Syst. (2019) 29:1850025. 10.1142/S0129065718500259 29954235

[35] Cao L Fan C Wang H Zhang G. A novel combination model of convolutional neural network and long short-term memory network for upper limb evaluation using kinect-based system. IEEE Access. (2019) 7:145227–34. 10.1109/ACCESS.2019.2944652

[36] Guo S Ding Y Guo J. Control of a lower limb exoskeleton robot by upper limb semg signal. 2021 In: IEEE International Conference on Mechatronics and Automation (ICMA). Takamatsu: IEEE (2021), p. 1113–1118.37535661

[37] Tjhai C O'Keefe K. Using step size and lower limb segment orientation from multiple low-cost wearable inertial/magnetic sensors for pedestrian navigation. Sensors. (2020) 19:3140. 10.3390/s19143140 31319508 PMC6679558

[38] Dutta A Sarkar K Tarun K. Machine learning regression algorithms for predicting the susceptibility of jointed rock slopes to planar failure. Earth Sci Inform. (2024) 17:2477–93. 10.1007/s12145-024-01296-5

[39] Lin S Zheng H Han C Han B Li W. Evaluation and prediction of slope stability using machine learning approaches. Front Struct Civil Eng. (2021) 15:821–33. 10.1007/s11709-021-0742-8

[40] Xie H Dong J Deng Y Dai Y. Prediction model of the slope angle of rocky slope stability based on random forest algorithm. Mathem Prob Eng. (2022) 2022:9441411. 10.1155/2022/9441411

[41] Ahangari Nanehkaran Y Pusatli T Chengyong J Chen J Cemiloglu A Azarafza M. Application of machine learning techniques for the estimation of the safety factor in slope stability analysis. Water. (2022) 14:3743. 10.3390/w14223743

[42] Asteris PG Rizal FIM Koopialipoor M Roussis PC Ferentinou M Armaghani DJ. Slope stability classification under seismic conditions using several tree-based intelligent techniques. Appl Sci. (2022) 12:1753. 10.3390/app12031753

[43] Yan Y Chen R Yang Z Ma Y Huang J Luo L. Application of back propagation neural network model optimized by particle swarm algorithm in predicting the risk of hypertension. J Clini Hypertens. (2022) 24:1606–17. 10.1111/jch.14597 36380516 PMC9731601

[44] Wu Y Xin B Wan Q Ren Y Jiang W. Risk factors and prediction models for cardiovascular complications of hypertension in older adults with machine learning: a cross-sectional study. Heliyon. (2024) 10:e27941. 10.1016/j.heliyon.2024.e27941 38509942 PMC10950703

[45] Han H. Diagnostic value of dynamic enhanced multi-slice spiral CT in lymph node metastasis of cervical cancer and analysis of the causes of missed diagnosis. Eur J Gynaecol Oncol. (2024) 45:6. 10.22514/ejgo.2024.006

[46] Gao J Liu Y. Prediction and the influencing factor study of colorectal cancer hospitalization costs in China based on machine learning-random forest and support vector regression: a retrospective study. Front Public Health. (2024) 12:1211220. 10.3389/fpubh.2024.1211220 38389946 PMC10881792

[47] Wu C Zha D Gao H. Prediction of bronchopneumonia inpatients' total hospitalization expenses based on BP neural network and support vector machine models. Comput Math Methods Med. (2024) 2022:9275801. 10.1155/2022/9275801 35633928 PMC9132643

[48] Sahoo AK Pramanik J Jayanthu S Samal AK. Slope stability predictions using machine learning techniques. In: 2022 4th International Conference on Advances in Computing, Communication Control and Networking (ICAC3N). Greater Noida: IEEE (2022). p. 133–137.

[49] Karir D Ray A Bharati AK Chaturvedi U Rai R Khandelwal M. Stability prediction of a natural and man-made slope using various machine learning algorithms. Transp Geotech. (2022) 34:100745. 10.1016/j.trgeo.2022.100745

[50] Khan MA Memon SA Farooq F Javed MF Aslam F Alyousef R. Compressive strength of fly-ash-based geopolymer concrete by gene expression programming and random forest. Adv Civil Eng. (2021) 2021:6618407. 10.1155/2021/6618407

[51] Fei H Fan Z Wang C Zhang N Wang T Chen R. Cotton classification method at the county scale based on multi-features and random forest feature selection algorithm and classifier. Remote Sensing. (2022) 14:829. 10.3390/rs14040829

